# Nasal endoscopy associated with paranasal sinus computerized tomography scan in the diagnosis of chronic nasal obstruction

**DOI:** 10.1016/S1808-8694(15)31336-7

**Published:** 2015-10-20

**Authors:** Aracely Fernandes Duarte, Rita de Cássia Soler, Francis Zavarezzi

**Affiliations:** ^1^ Resident Physician, Service of Otorhinolaryngology, Hospital Nossa Senhora de Lourdes/SP; ^2^ Ph.D. in Otorhinolaryngology, Faculdade de Ciências Médica da Santa Casa de São Paulo, Preceptor of Residence/Internship Service in Otorhinolaryngology, Hospital Nossa Senhora de Lourdes; ^3^ Resident Physician in Otorhinolaryngology, Hospital Nossa Senhora de Lourdes

**Keywords:** nasal endoscopy, computer tomography scan, nasal obstruction

## Abstract

**C**hronic nasal obstruction is a common complaint in Otolaryngology outpatients. The diagnosis of nasal obstruction is based on the clinical history, physical examination and diagnostic procedures. Among these, it is already established in the current literature the importance of nasal endoscopy and computer tomography scan. **Aim**: The objective of this research study was based on a comparative study among findings of nasal endoscopy and CT scan of the paranasal sinuses, within the examinations for etiological investigation in chronic nasal obstruction, individualizing the importance of each exam for a conclusive diagnosis. **Study design**: Historic cohort. **Material and Method**: Twenty patients with chronic nasal obstruction complaints were studied, aged between 14 and 51 years old in the Otolaryngology outpatient unit at Nossa Senhora de Lourdes Hospital, Sao Paulo. It is a retrospective clinical study, carried out by revision of medical charts of assisted patients from 2002 to 2004. **Results**: All the patients presented complaints of chronic nasal obstruction. In the 20 patients, 10 (50%) presented associated allergic complaints. In 16 out of 20 (80%), patients presented hypertrophic concha evidenced by nasal endoscopy; in only 9 out of 20 (45%) patients we found the same affection as in the CT scan. Based on the presented results, the finding of hypertrophic concha was more evidenced in nasal endoscopy compared to CT (80% X 45%). Two cases of nasal polyposis were evidenced in nasal endoscopy but not in CT, besides two other cases without detection in the CT, but detected by nasal endoscopy, in other words, normal CT with abnormal nasal endoscopy. **Conclusion**: Thus, the presented study and the results of nasal fossa findings obtained by nasal endoscopy were more conclusive in the elucidation of diagnosis than those obtained by computer tomography of the paranasal sinus.

## INTRODUCTION

Chronic nasal obstruction is a common complaint in Otorhinolaryngology outpatient practice.

The diagnosis of nasal obstruction is based on clinical history of nasal disease and physical examination. However, many complementary tests are required in some cases. Among them, the current literature has already confirmed the importance of nasosinusal endoscopy and computed tomography scan.

Nasosinusal endoscopy is mentioned as a standard test to precisely assess nasal obstructive disease and it is considered necessary in all patients with nasal obstruction, especially after the second week of evolution.

Computed tomography scan (CT) of the nose and paranasal sinuses is the ideal imaging exam (gold standard) to study nasal and paranasal sinuses diseases. It has high sensitivity because it provides precise information about soft and bone parts of the nasal cavity, paranasal sinuses, orbit and endocranium. It is an exam performed at axial and coronal sections and it allows saggital reconstructions with the use of intravenous contrast, when necessary.

## OBJECTIVES

The purpose of the present study was to carry out a comparative analysis of the findings of nasosinusal endoscopy and paranasal sinuses CT scan within the etiological investigation of chronic nasal obstruction, individually defining the importance of each exam for diagnostic conclusion.

## MATERIAL AND METHOD

We studied 20 patients with complaint of chronic nasal obstruction, ages ranging from 14 to 51 years, seen in the Outpatient Service of Otorhinolaryngology, Hospital Nossa Senhora de Lourdes, Sao Paulo. It is a clinical retrospective study carried out by reviewing medical charts of patients seen between 2002 and 2004.

## RESULTS

All patients presented complaints of chronic nasal obstruction. Out of 20 patients, 10 (50%) presented associated allergic complaints. Out of the total, 10 (50%) patients had chronic nasal obstruction as a single complaint; 8 (40%) had associated headache, and 2 (10%) had complaints of pharyngeal bolus associated with nasal obstruction.

In 16 out of 20 patients (80%) we found turbinate hypertrophy evidenced by nasofibroscopy; only 9 out of 20 patients showed the same affection at CT scan.

In 2 patients (10%) we found nasal polyposis at nasofibroscopy, but the same affection was not found in the CT scan. In 17 patients (85%), we observed septal deviation and 8 of them presented it at nasofibroscopy and in CT scan (47.1%), in 3 only at nasofibroscopy (11%), and in 6 patients septal deviation was found by CT scan alone (22%).

In 2 cases in which CT scan was normal, nasofibroscopy presented abnormalities, which were isolated septum deviation in one case and septal deviation associated with turbinate hypertrophy in the other case.

At the level of the nasal fossae, anatomical affections found in the exams of the studied patients were mainly lower turbinate hypertrophy and septal deviation. We also found bullous middle concha (20%), medium concha with curvature inversion (5%) and nasal polyposis (10%).

## DISCUSSION

According to Castagno, 1993, many different factors contribute to nasal cavity obstructions, from viral, allergic processes to septal deviations, turbinate hypertrophy and other anatomical and intranasal abnormalities. The 20 patients with clinical picture of chronic nasal obstruction in our study had structural anatomical affections, which were pointed as cause of the complaint made.

Based on the presented results, the finding of turbinate hypertrophy was more evidenced by nasofibroscopy than by CT scan (80% × 45%). Two cases of nasal polyposis were evidenced by nasofibroscopy and not by CT scan, in addition to two other cases in which we did not observe CT affections, which were detected by nasofibroscopy, that us, normal CT scan with nasofibroscopy affections (septum deviation and turbinate hypertrophy).

Similar findings were reported by Vining, 1993, in which patients who had negative CT scan showed endoscopic exam with septal deviation, mucosa edema involving the middle meatus, as well as adenoid and turbinate hypertrophy.

**Chart 1:**
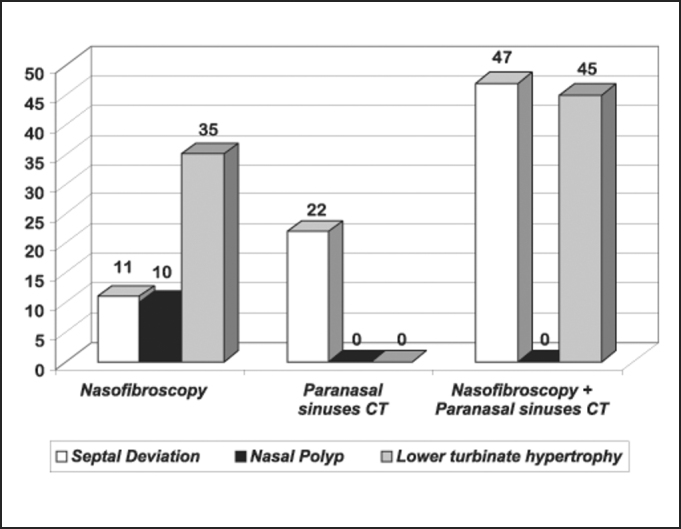
Distribution of findings by performed exam.

Pizzichetta, 1994, in a similar study, did not consider the CT findings in most of the studied cases to explain the symptoms of nasal obstruction, considering the endoscopic exam enough to that purpose.

Nasosinusal endoscopy and CT scan can be considered complementary techniques for effective demonstration of nasal anatomy and paranasal sinuses, according to Morra (1998). Such statement is added to the theory that the CT scan would be more specific for the assessment of paranasal sinuses, whereas nasosinusal endoscopy would have better resolubility to assess nasal fossa, according to Tratado (2003). Thus, the results of our present study confirmed the existing literature.

## CONCLUSION

Therefore, in view of the present study and the results obtained we could observe that as far as the paranasal sinuses go, findings obtained by nasosinusal endoscopy were more conclusive for diagnostic elucidation than those obtained for paranasal sinuses computed tomography, taking into account, however, that the technical aspects required for the CT scan sections could have influenced the results of the present study.
